# Genomic profiling at a single center cracks the code in inborn errors of immunity

**DOI:** 10.1007/s11739-025-03871-0

**Published:** 2025-01-28

**Authors:** Alessandro Andriano, Vanessa Desantis, Carolina Marasco, Antonio Marzollo, Silvia Bresolin, Nicoletta Resta, Lucia Di Marzo, Fabrizio Pappagallo, Antonella Mascolo, Ingrid Catalina Caradonna, Simona D’Amore, Angelo Vacca, Antonio Giovanni Solimando

**Affiliations:** 1 Pharmacology Section, Department of Precision and Regenerative Medicine and Ionian Area (DiMePRe-J), University of Bari Aldo Moro Medical School, Bari, Italy; 2 Unit of Internal Medicine and Clinical Oncology “G. Baccelli”, Department of Precision and Regenerative Medicine and Ionian Area (DiMePRe-J), University of Bari Aldo Moro Medical School, Bari, Italy; 3 Pediatric Hematology, Oncology and Stem Cell Transplant Division, Padua University Hospital, Padua, Italy; 4 Onco-Hematology, Stem Cell Transplant and Gene Therapy, Istituto Di Ricerca Pediatrica Foundation - Città Della Speranza, Padua, Italy; 5 Medical Genetic, Department of Precision and Regenerative Medicine and Ionian Area (DiMePRe-J), University of Bari Aldo Moro, Bari, Italy

**Keywords:** IEI, NGS, TGP, CARMIL2, TACI, STAT3

## Abstract

Inborn errors of immunity (IEI) entail a diverse group of disorders resulting from hereditary or de novo mutations in single genes, leading to immune dysregulation. This study explores the clinical utility of next-generation sequencing (NGS) techniques in diagnosing monogenic immune defects. Eight patients attending the immunodeficiency clinic and with unclassified antibody deficiency were included in the analysis. Clinical records, immune characteristics, and family histories were reviewed, and a target gene panel (TGP) sequencing was performed to identify pathogenic variants. TGPs identified seven variants in *TNFRSF13B* (TACI), *CARMIL2*, *STAT1*, *STAT3*, and *ORAI1* genes. These findings provided definitive diagnoses and proper prognostic assessment. Patients exhibited a wide range of clinical manifestations, including recurrent infections, autoimmune cytopenias, and organ-specific complications. The genetic diversity observed highlights the importance of genetic testing in diagnosing IEIs and tailoring treatments. This study underscores the role of TGPs in diagnosing IEIs, revealing significant genetic heterogeneity and phenotypic variability. They offer a precise tool for identifying underlying genetic defects, facilitating personalized medicine approaches, and eventually improving patient outcomes. The findings emphasize the need for comprehensive genetic testing to uncover novel pathogenic variants, enhancing our understanding of immune system dysfunction. NGS is a critical tool for the management of IEI, enabling precise diagnosis and personalized treatment strategies. Despite resource limitations, the progressive affordability is likely to expand its clinical utility, ultimately improving patient care and advancing the field of immunology. In the meantime, accurate phenotypic assessment is essential for resource optimization and case prioritization.

## Introduction

Inborn errors of immunity (IEI) are a heterogeneous group of diseases driven by genetic defects in immune regulation, which exhibit a wide spectrum of clinical manifestations, including inflammatory states, autoimmunity, and increased susceptibility to specific pathogens. The prevalence of these conditions is estimated to be approximately 1 in 1000 to 5 in 1000 [1]. Advances in the understanding of the molecular and immunological mechanisms behind these diseases [2] previously classified as primary immunodeficiency (PID) have prompted different phenotype-based approaches mainly focusing on autoimmunity and inflammatory manifestations that were previously underestimated, aimed to improve patient diagnosis and treatment [3]. In this view, a convenient classification based on genotype–phenotype correlation was developed by the International Union of Immunological Societies Committee in 2022 [4]. The IUIS Committee also provided phenotype-based tables to guide clinicians and lead them to the proper diagnostic work-up [5]. However, even this approach is limited due to the phenotypic variability of different gene variants and overlapping phenotypes that prevent a straightforward association. Moreover, different variants of the same gene may have similar onsets but completely different prognoses, thus requiring proper management and a tailored therapeutic approach. As a matter of fact, in most cases, a definite diagnosis cannot be solely achieved on a clinical or laboratory basis but stems from genetic testing as only definite confirmation. As a consequence, patients with IEIs face diagnostic and treatment delays. Next-generation sequencing (NGS) techniques have progressively spread in clinical settings, thanks to their increased affordability; the use of NGS has allowed the identification of an increasing number of monogenic causes of IEIs, thus revealing novel genes with specific immune functions as well as critical roles of known genes not previously associated with immune regulation, ultimately enabling more targeted approaches. In recent years, the cost-effective target gene panels (TGPs) and whole-exome sequencing (WES) have already proven their role in prompt diagnosis, treatment plan and prognosis as well as in the discovery of novel pathogenic variants [6]. However, the lack of resources still prevents systematic sequencing for all eligible patients.

Among IEI, common variable immunodeficiency (CVID) remains the most prevalent phenotype falling into the category of predominantly humoral defects. Clinical diagnostic criteria underwent several revisions without reaching a worldwide consensus and are still under debate [7]. As a result, the term CVID is controversial and diagnosis of the disease is mostly established by exclusion despite extensive ongoing efforts to identify pathognomonic clinical characteristics or laboratory analyses. On the other hand, these analyses are notably not available in every center and results should still be monitored over time [5, 8]. In this study, we evaluated the clinical utility of TGP sequencing and its impact on both prognosis and different therapeutic approaches in a subset of patients with unclassified antibody deficiency and for whom a monogenic form was suspected based on the presence of syndromic features along with severe hypogammaglobulinemia or signs of immune dysregulation in the form of autoimmunity or inflammatory disease. Our overarching goal was to advance the knowledge in the field of clinical immunology by increasing awareness of genetic testing value in identifying gene defects involved in immune diseases and promoting optimal management and targeted treatment where available.

## Methods

### Patient selection

**Fig. 1:**
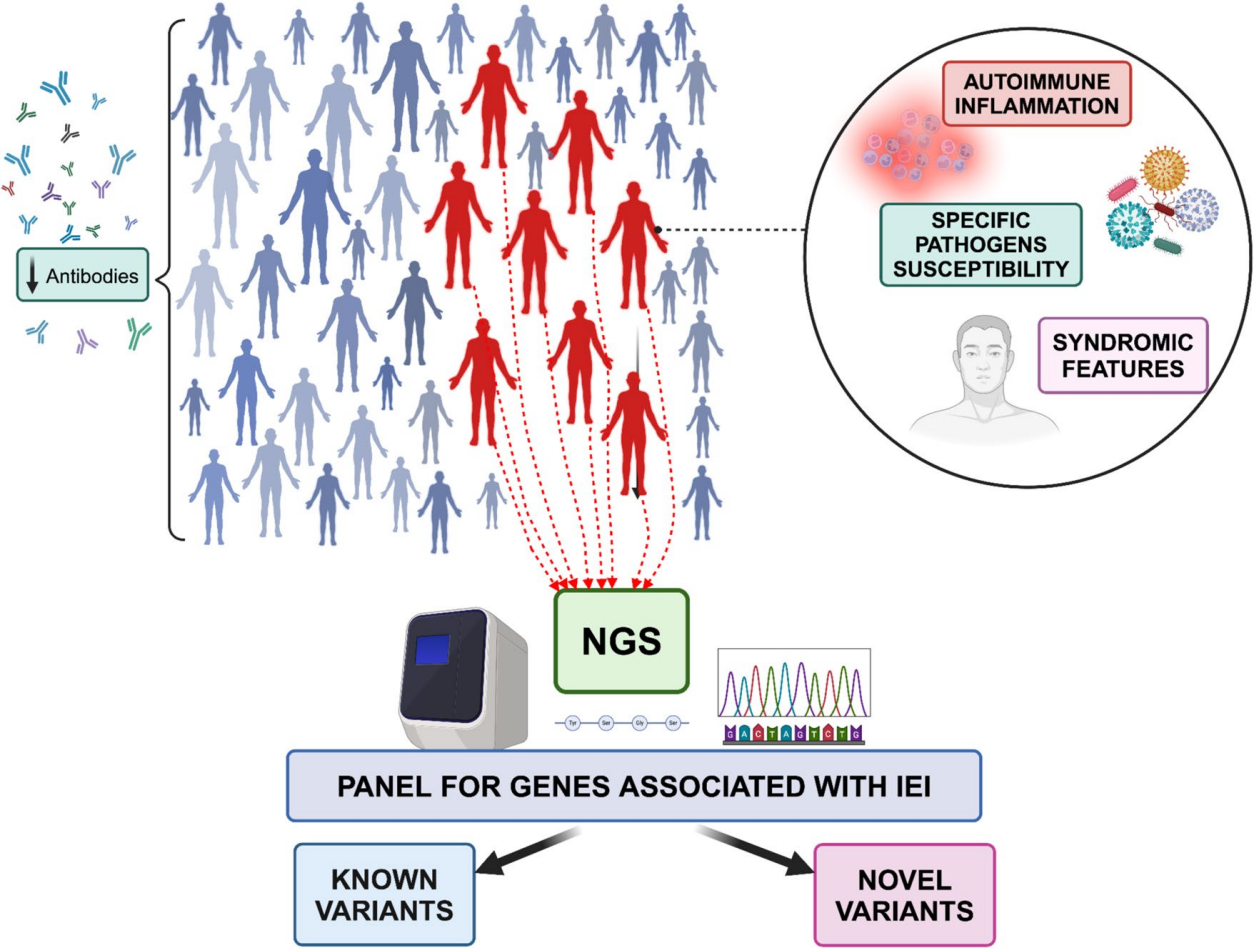
Graphical summary of methods

Between March 2022 and March 2023, clinical and laboratory records, immune characteristics and family history of 52 consecutive patients with an old diagnosis of CVID and attending the Immunodeficiency Clinic at the University of Bari Hospital (Italy) were reviewed according to current indications and recent literature revisions [4, 5, 9]. All patients met the criteria for unclassified antibody deficiency (UAD) according to the European Society of Immunodeficiency (ESID) criteria [9] and were routinely receiving immunoglobulins replacement therapy (IgRT). In our cohort, 8 subjects who displayed syndromic features, a clinical phenotype of immune dysregulation, namely autoimmunity or inflammatory disease, and/or susceptibility to atypical infections, such as opportunistic infections were selected for genetic testing (Fig. 1).

Figure 1 Phenotype-Based Approach for Identifying Genetic Variants: A schematic representation of methods employed in this study to uncover genetic variants. Records of patients with CVID-like phenotype were reviewed according to the presence of syndromic features, autoimmune inflammation, and susceptibility to specific infections. Patients that met the aforementioned criteria were selected for NGS genetic testing. Created in BioRender. Solimando, A. (2025) https://BioRender.com/t15u327.

This study was conducted in conformity with the European Directive on Good Clinical Practice and the ethical guidelines of the Declaration of Helsinki (as revised and amended in 2004), with the approval of the Ethics Committee of the University of Bari Medical School (studies n° 900, 31/08/2022 and n°587, 20/06/2023). Participation in the study was voluntary and all enrolled patients provided their informed consent. The main laboratory findings of selected patients are summarized in Table 1.

**Table 1:** Immunoglobulin levels and standard immune phenotype characterization

Case	IgG (mg/dl)	IgA (mg/dl)	IgM (mg/dl)	Total lymphocytes number (cells/µL)	B Cell (%)	NK Cell (%)	T Cell (%)	CD8^+^ (%)	CD4^+^ (%)
#1	1006	5	305	4370	4	5	90	41	39
#2	690	8	26	258	5	10	85	15	70
#3	1004	63	33	1798	9	7	85	35	46
#4	740	278	123	3384	21	1	77	17	56
#5	940	325	200	2504	11	1	88	20	66
#6	320	8	5	1427	5	2	92	48	38
#7	850	8	10	1084	0	1	98	71	18
#8	717	1072	5	573	9	17	74	41	29

All Patients were receiving immunoglobulin replacement therapy (IgRT)

### Sequencing

Call for variants bioinformatic analysis was performed using the BWA-MEM system (v. 0.7.17), software GATK4 Haplotype Caller (v. 4.0.6.0), while their notation was performed using snpEff (v.4.3t) e ANNOVAR. Interpretation of variants based on clinical indication relies on the following databases: GnomAD, SIFT, PolyPhen, CADD, and ClinVar. Variants were evaluated according to ACMG [10] and reported as HGVS notation (www.hgvs.org/varnomen). Poor-quality variants were excluded (coverage < 10X or alternative transcript VAF < 30%). Variants covered between 10 and 20X were considered only if they were closely related to the clinical phenotype. All identified variants were confirmed by Sanger sequencing. Data analysis was performed by CodonCode Aligner and DNASTAR Lasergene Softwares. To characterize structural rearrangements, we used the Infinium CytoSNP-850 K v1.2 BeadChip, which includes approximately 850,000 markers for detecting copy number variations, such as microdeletions, microduplications, and regions of homozygosity. Genomic DNA was extracted from peripheral blood, quantified, and processed following the manufacturer's protocol.

## Results

Clinical cases are presented below with sequencing results. Clinical features are summarized in Table 2, sequencing results are reported in Table 3.

**Table 2:** Summary of clinical records revision

Case	Sex	Age (years)	Phenotype onset (months)	Diagnosis (months)	Main clinical features
#1	M	57	24	36	Autoimmune hemolytic anemia, immune thrombocytopenia, inflammatory bowel disease, recurrent infections by *Streptococcus pneumoniae and Neisseria meningitidis*, splenomegaly, splenic vein thrombosis, rectal adenocarcinoma
#2	F	46	216	411	Recurrent infections, bronchiectasis, autoimmune hemolytic anemia, immune thrombocytopenia, hepatosplenomegaly, hepatic fibrosis, portal hypertension, and inflammatory bowel disease
#3	F	22	72	212	Interatrial defect, recurrent infections, atrophic gastritis, not-specific colitis, severe underweight
#4*	M	44^✝^	36	36	Bronchiectasis, palmoplantar psoriasis, CMV infections, ulcerative eosinophilic gastro-esophagitis, hypertrophic cardiomyopathy
#5*	M	38	6	18	Bronchiectasis, nail psoriasis, tuberculosis infection, oral leukoplakia, *molluscum contagiosum*, gastric ulcers, hypertrophic cardiomyopathy
#6	M	31	72	288	Chronic sinusitis, splenomegaly, infectious manifestations, gastrointestinal inflammation, acute bronchitis
#7	F	33^✝^	6	283	Mucocutaneous candidiasis, opportunistic pulmonary infections, type 1 diabetes, pancytopenia
#8	F	28	12	60	Clubfoot, cleft palate, pancytopenia, reduced C3-C4, EBV positivity, hepatomegaly, recurrent VZV infections

^*^Siblings

^✝^Deceased

**Table 3:** Identified variants in the selected patients

Case	Gene	Accession	Nucleotide	Amino acid	Zygosity (VAF)	Classification	Novel (?)	Disease (MIM#)	Inheritance
#1	TNFRSF13B	NM_012452.3	c.310 T > C	p.(Cys104Arg)	Heteroz	Risk Factor	Known	CVID (240,500)	AD
#2	TNFRSF13B	NM_012452.3	c.260 T > A	p.(Ile87Asn)	Heteroz	Risk Factor	Known (rare)	CVID (240,500)	AD/AR
	TNFRSF13B	NM_012452.3	c.310 T > C	p.(Cys104Arg)	Heteroz	Risk Factor	Known	CVID (240,500)	AD/AR
#3	TNFRSF13B	NM_012452.3	c.260 T > A	p.(Ile87Asn)	Heteroz	Risk Factor	Known	CVID (240,500)	AD/AR
#4	CARMIL2	NM_001013838.3	c.993_994 insA	p.(Ala332AspfsTer30)	Homoz	Likely pathogenetic	Novel	–	–
	IL36RN	NM_012275.3	c.335dup	p.(Ser113ValfsTer14)	Heteroz	Likely pathogenetic	Novel	Pustular Psoriasis (614,204)	–
			c.338C > T	p.(Ser113Leu)	Heteroz	Likely pathogenic	Known (rare)	Pustular Psoriasis (614,204)	AR
	MYBPC3	NM_000256.3	c.2429G > A	p.(Arg810His)	Heteroz	Likely pathogenetic	Known	Hypertrophic Cardiomyopathy (115,197)	AD
#5	CARMIL2	NM_001013838.3	c.993_994 insA	p.(Ala332AspfsTer30)	Homoz	Likely pathogenetic	Novel	–	–
	IL36RN	NM_012275.3	c.335dup	p.(Ser113ValfsTer14)	Heteroz	Likely pathogenetic	Novel	Pustular Psoriasis (614,204)	–
#6	STAT3	NM_139276.3	c.1333G > T	p.(Val445Lys)	Heteroz	Under evaluation	Novel	–	–
#7	STAT1	NM_007315.4	c.520 T > C	p.(Cys174Arg)	Heteroz	Likely pathogenetic	Novel	Immunodeficiency 31C (614,162)	–
#8	ORAI1	NM_032790	Microdeletion of 43.8 kb in region 12q24.31		–	Conflicting interpretations	–	Immunodeficiency (612,762)–Myopathy (615,883)	AR–AD

### Patient#1

A 20-year-old male with a history of recurrent infections developed autoimmune hemolytic anemia that was successfully treated with corticosteroid therapy. Aged 43 years, he had an immune thrombocytopenic purpura, which required prolonged treatment with immunosuppressants and corticosteroids. In the same year, he underwent a resection of a rectal villous adenocarcinoma. A year later, hypogammaglobulinemia was found. He started intravenous IgRT considering his history of infections. Four years later, a follow-up colonoscopy revealed a non-specific inflammatory state with eosinophilic and neutrophilic infiltration. Gastroscopy accounted for gastric congestion and esophageal varices. In the same year, a CT scan showed multiple lymphadenopathies, hepatosplenomegaly and renal hematoma. For two consecutive years, he had two different episodes of hemolytic anemia that required corticosteroid treatment. Splenic vein thrombosis posed the indication for a splenectomy, a cholecystectomy was performed too. Aged 52 years old, he was admitted to the emergency department for tetraparesis due to *Streptococcus pneumoniae* meningitis. During this admission, a bone marrow biopsy was performed that showed a hypercellular marrow with myeloid/erythroid ratio of 1:1. His last CT scan showed hepatic nodular hyperplasia. Genetic testing identified a known TNFRSF13B variant (p.Cys104Arg) coding for TACI receptor, known to be associated with CVID. TACI mutations are not per se causative of the disease; however, certain TNFRSF13B mutations can predispose CVID patients to autoimmunity and lymphoproliferation [11].

### Patient#2

An 18-year-old pregnant woman with an occasional finding of thrombocytopenia and hypogammaglobulinemia at the 20th week of gestation was efficiently treated with corticosteroid therapy. Aged 33 years, she performed a CT scan to investigate recurrent lower airways infections unresponsive to conventional antibiotic therapy. The CT scan revealed multiple bronchiectasis at the right middle lobe that were surgically resected. She started IgRT. Three years later, she developed hepatosplenomegaly and portal hypertension. At the age of 38 years, she had refractory hemolytic anemia with a positive direct antiglobulin test and IgM C3c C3d autoantibodies. Because of irregular bowel habits, she underwent a colonoscopy that showed non-specific inflammation with plasma cells, neutrophils and eosinophils infiltration as well as cryptitis and abscesses. She had a spleen infarction that led to splenectomy. Aged 43 years, she showed esophageal varices and gastric congestion. A CT scan revealed advanced hepatic fibrosis and retroperitoneal lymphadenopathy. Moreover, histological samples collected during a follow-up colonoscopy revealed focal lymphatic hyperplasia and a chronic inflammatory state implying ulcerative colitis. Genetic sequencing revealed bi-allelic TNFRSF13B high-risk variants (p.Cys104Arg and p.Ile87Asn).

### Patient#3

A female, born after Cesarean section, showed an interatrial defect with a normal hemodynamic state. Since preschool, she had respiratory, gastrointestinal, and urinary recurrent infections. At six years old, she was hospitalized for acute gastroenteritis. Five years later, she was hospitalized for acute conjunctivitis. Aged 15 years, she showed hypogammaglobulinemia at serum protein electrophoresis. Two years later, she started IgRT. When she was eighteen years old, a gastroscopy revealed signs of atrophic gastritis. She tested negative for Helicobacter pylori. She underwent a colonoscopy which revealed not-specific colitis. She claims she met all growing milestones, but now she appears significantly underweight. TGP sequencing identified a rare TNFRSF13B variant (p.Ile87Asn), confirming its role in TACI-associated immunodeficiencies.

### Patient#4 and patient#5

A 3-year-old male was diagnosed with CVID. At a young age, he was already suffering from recurrent infections, palmoplantar psoriasis, and bronchiectasis surgically treated with lobectomy. Before reaching his forties, he developed recurrent cytomegalovirus (CMV) infections, compromising his sight, and ulcerative eosinophilic gastro-esophagitis. Later, he developed chronic axonal neuropathy and an ulcerative and obstructive lesion at the ileocecal valve. He was found to show hypertrophic cardiomyopathy.

**Fig. 2:**
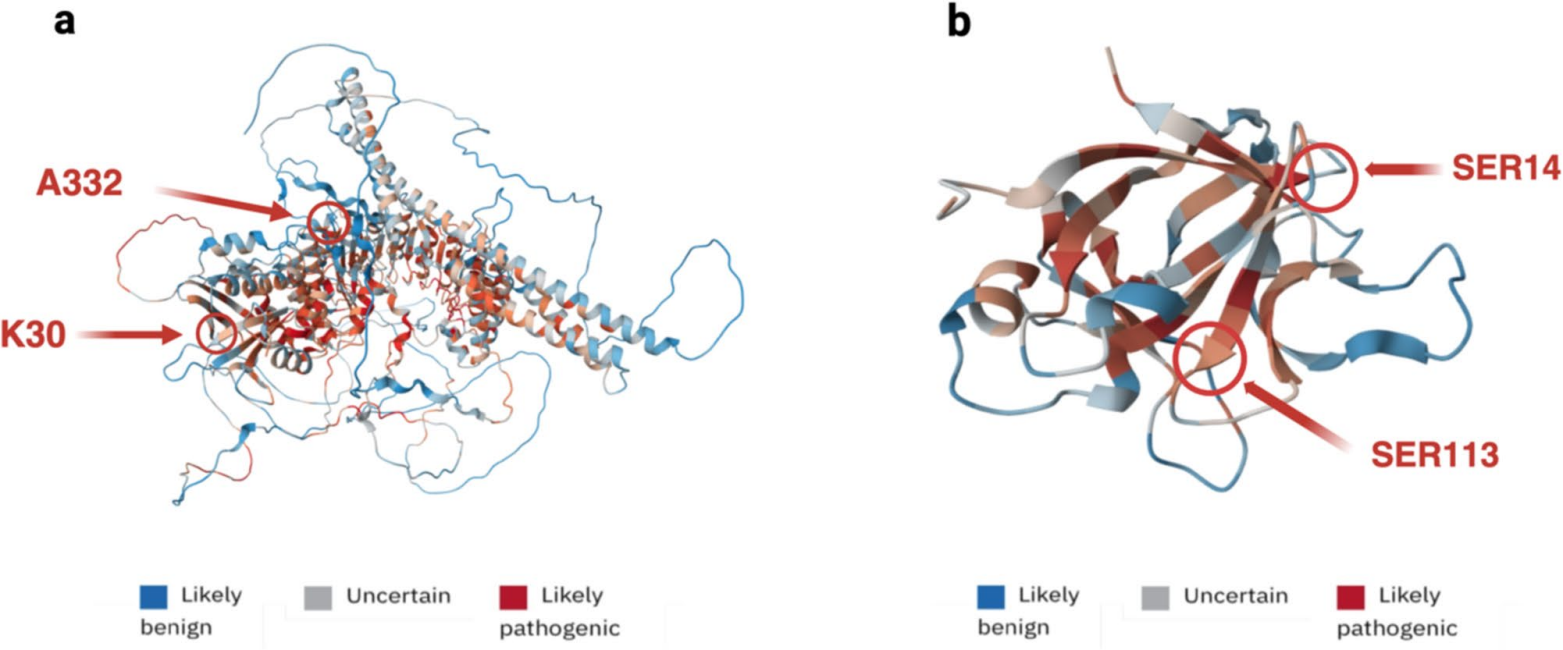
CARMIL2 and IL36RN protein product structure underlying the identified novel variants sites

His seven-year-old younger brother was closely followed up and diagnosed with a CVID too. He readily developed psoriasis too, mainly with ungual and cutaneous manifestations. At about 20 years of age, he experienced a tuberculosis infection and a spontaneous pneumothorax. His immunophenotype showed normal counts of B cells, both switched and not switched. T-cell receptor excision circles (TRECs) analysis was negative. In his thirties, he developed oral leukoplakia on two occasions that was surgically removed. His CT scan was found positive for pulmonary bronchiectasis. Later, he developed periocular and forearm lesions by molluscum contagiosum and consistently showed reduced levels of cytotoxic T lymphocytes and natural killer cells, and increased levels of T helper lymphocytes. He also has low levels of vitamin B12, and an esophagogastroduodenoscopy showed gastric ulcers. NGS revealed a novel CARMIL2 homozygous variant that is likely pathogenic (c.993_994insA, p.Ala332AspfsTer30). The two siblings were found to be positive also for a known pathogenic variant IL36RN gene, with the older one showing even another variant in trans position. IL36RN gene mutations are a well-known cause of pustular psoriasis by increasing IL-6 production and offer a chance for target therapy of the skin disease [4, 12]. The structure of protein products of those genes is shown in Fig. 2. Both patients were offered a hematopoietic stem cell transplant (HSCT): unfortunately, the older patient died because of infections, while the younger brother is currently a candidate for HSCT. Perhaps, phenotype of the older sibling could be complicated by the additional pathogenic variant in IL36RN gene.

Figure 2 CARMIL2 (a) and IL36RN (b) protein structure prediction and pathogenicity for possible missense mutations. The main amino acid positions involved in the identified mutations are shown. The CARMIL2 (a) identified novel variant is characterized by frameshift mutations starting from the insertion of adenine that results in alanine substitution for aspartate in position 332 and ending with chain termination in position 30. Both the N-terminal and the leucine repeat-rich (LRR) domain, essential for protein interactions, are altered [13]. Given the tremendous modification, the protein product of this CARMIL2 variant is very likely to cause a loss-of-function (LOF). The IL36RN (b) identified novel variant is characterized by frameshift mutations starting with serine substitution for valine in position 113 and ending with chain termination in position 14. Intensity of color relates to risk for missense mutation pathogenicity in each position as coded in the figure legend [14, 15]. Modified with BioRender.com.

### Patient#6

A 25-year-old male presented with chronic rhinitis, splenomegaly, and irregular bowel habits. He was hospitalized due to acute tonsillitis at the age of 6 years, then again because of tenosynovitis of the right-hand second finger. He had a history of recurrent sinus infections and acute bronchitis. His immunoglobulin levels were below the lower limit of normal range and an immunophenotypic analysis showed low levels of switched memory B cells and increased levels of unswitched memory B cells. KREC functional analysis confirmed B cell dysfunction. He was diagnosed with CVID. Endoscopic examination of gastrointestinal tract, followed by histological analysis, revealed the presence of chronic gastritis, intestinal nodular hyperplasia, with increased levels of duodenal intraepithelial T lymphocytes, cryptitis with eosinophilic and neutrophilic infiltration, and absence of plasma cells. Results were not consistent with inflammatory bowel disease. HLA characterization ruled out a predisposition to celiac disease. TGP sequencing revealed the presence of an undescribed variant (c.1333G > T:p.V445L) of uncertain significance in STAT3 gene. His clinical presentation was characterized by intestinal chronic inflammation and hypogammaglobulinemia, suggesting a STAT3 GOF rather than a LOF. Gluten-free diet seemed to improve patient’s gastrointestinal condition in discordance with HLA testing. IgRT was confirmed without evidence of the need for target therapy.

**Fig. 3:**
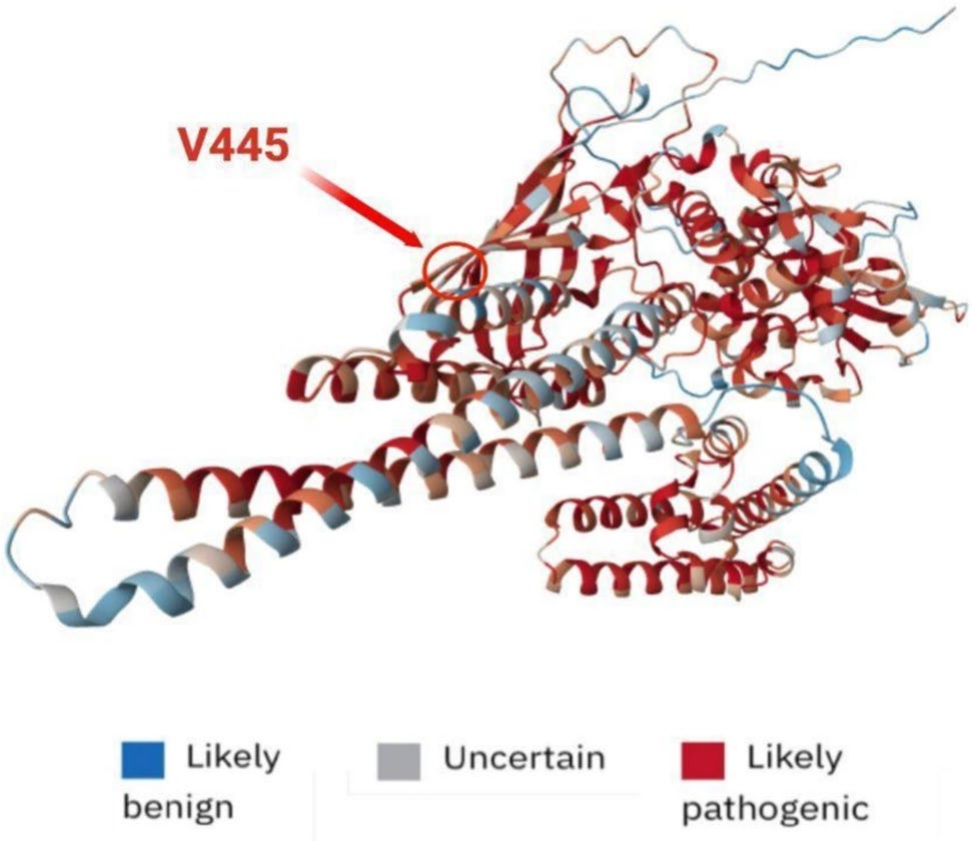
STAT3 structural model and missense mutation pathogenicity prediction

Figure 3 STAT3 protein structure prediction and prediction of pathogenicity for possible missense mutations. Investigation on polyphen2 [16] for prediction of missense mutation gave negative results for possible pathogenicity. Contrarily, results from the analysis on Alphafold platform considered missense mutation for Valine in position 445 at high risk for pathogenicity. Further experimental analyses are required for accurate assessment and characterization of this novel STAT3 variant (average Alphamissense pathogenicity score: 0.877) [14, 15]. Intensity of color relates to risk for missense mutation pathogenicity in each position as coded in the figure legend. Modified with BioRender.com.

### Patient#7

A 32-year-old female was admitted with pallor, weakness, cough, and dyspnea. She was pale but well-appearing with a body mass index (BMI) of 19,5 kg/m^2^. She had painful oral ulcers, a history of oral candidiasis, recurrent pneumonia, and type 1 diabetes mellitus (T1DM). Her condition worsened rapidly in the two weeks after admission and her laboratory tests showed pancytopenia. Although the PLT count remained relatively preserved, PB neutrophils < 500/mL, reticulocytes < 2%, and BM hypocellularity confirmed the diagnosis of severe aplastic anemia. The pedigree chart suggested an autosomal-dominant inheritance pattern for the T1DM phenotype with severe oral and esophageal candidiasis [17]. Genetic sequencing identified a heterozygous variant (p.520 T > C, p.Cys174Arg, rs387906763) of STAT1 and the patient was offered HSCT. Compassionate use of JAK/STAT inhibitors was also proposed in the peri-transplant period; however, the patient died after a JC virus infection, before starting treatment. The case has been previously reported [17], and it represents one of few reports of a STAT1 mutation associated with bone marrow failure [18].

### Patient#8

A female was born at the 38th week of gestation with a cleft palate and bilateral clubfoot. Aged 2 years, she underwent surgical correction. Karyotyping revealed the presence of a ring chromosome 18. Delayed physical and behavioral development milestones were reported. Starting from the age of ten, she suffered recurrent lower respiratory tract infections. Aged 15 years, she showed pancytopenia and hypogammaglobulinemia and was started on IgRT. At the age of 20 years, she underwent an endoscopic resection of five gastric polyps whose histological examination revealed the presence of only lympho-monocytic cells. By the age of 24 years, her laboratory tests showed reduced levels of C3 and C4 complement fractions, and an abdominal ultrasound scan confirmed the presence of splenomegaly. She experienced recurrent episodes of Herpes Zoster infection. She later developed hepatomegaly; she underwent a bone marrow biopsy that showed hypercellular marrow and the presence of a 2% lymphocytic population. The clinical characteristics of selected patients are summarized in Table 2. TGP sequencing identified a ring chromosome 18 anomaly combined with an ORAI1 microdeletion explaining the immune defects. ORAI1 deficiency underscores the non-redundant role of calcium influx in lymphocyte function. Although immune defects are evident, therapies remain limited to symptomatic and supportive care.

## Discussion

IEIs are a group of rare disorders characterized by an increased susceptibility to infections, autoimmunity, hematological and solid malignancies that pose a diagnostic challenge due to their genetic, mechanistic and clinical heterogeneity. As a result, the accurate identification of disease-causing variants has not only the potential to elucidate the genetic basis underlying a phenotype, but it is also crucial for a more appropriate patient’s management and treatment. In this context, genetic testing offers a chance to assess the presence of a monogenic form of disease confirming the diagnosis IEI and allowing reclassification of the disease into another newly described category [4]. However, about 70% of cases remain without a genetic cause even after sequencing [6]. This underscores the need for the diffusion and implementation of NGS in clinical settings to confirm diagnosis and search for novel gene variants. Indeed, once the presence of a monogenic form confirms the diagnosis of IEI, reports on same or similar genetic variants allow a better prognostic assessment. In some cases, identifying pathogenic mutations offers a chance for target therapy and, more rarely, even curative gene therapy. Nevertheless, in most IEIs, as for those classified as severe combined immunodeficiency (SCID), HSCT is the only option available to reach complete cure even though its role in adult patients is still controversial and requires complex selection, evaluation of possible target therapy, as a bridge to transplant, and optimal timing [19, 20]. This emphasizes even more the importance of genetic testing for prompt diagnosis since the earlier the age of transplant, the greater are the chances for complete cure of immune defects [19, 21].

According to updated reports, patients with TACI variants usually exhibit recurrent infections, autoimmune cytopenias, and organ-specific complications. About 11% of CVID patients carry TACI mutations which influence the disease hallmark but do not cause it [22]. Interestingly, patient#2 has pathogenic variants in both alleles, but her phenotype is not significantly different. This result contrasts with the evidence that bi-allelic variants have a lower risk for autoimmune disorders [22, 23]. Moreover, at least 10% of CVID present with liver disease manifesting with only elevated levels of alkaline phosphatase (ALP), nodular regenerative hyperplasia or fibrosis/cirrhosis. Contributors are found in the other manifestations of the disease, namely recurrent infections, autoimmune disorders, lymphoproliferation, malignancy, granulomas, inflammatory infiltration, intrahepatic biliary obstruction, liver fibrosis, splenomegaly, and lymphocytic enteropathy [24]. Patient#3 did not show signs of hepatic involvement, but her younger age could simply suggest that chronic inflammation and lymphoid proliferation require more time to cause liver disease. Early detection of liver involvement is essential for proper prognostic evaluation and follow-up [25, 26]. Genetic testing can help identify those patients with higher risks as we proved the opposite to be also true, with liver disease being suggestive of a monogenic form. Preventive measures can be set in place, but unfortunately, no specific treatment is available yet. Different therapeutic strategies have been proposed by the use of immunomodulatory drugs, a.k.a. rituximab and infliximab, especially in the presence of nodular regenerative hyperplasia or granulomatous disease [27, 28]. In some cases, patients can be candidates for liver transplant, while there are only a few reports on allogeneic peripheral stem cell transplantation [24]. Splenectomy alone does not seem to reduce risk for liver disease [29]. TACI-based targeted therapy has recently been explored in the context of other immune diseases [30] and perhaps its role could be explored in TACI-associated liver disease. Further, investigations are necessary to find possible therapeutic strategies for reducing the disease burden in this subclass of patients with increased mortality [31].

CARMIL2 deficiency causes defective CD28-mediated T cell co-stimulation, altered cytoskeletal dynamics, susceptibility to various infections and an Epstein–Barr virus-induced smooth muscle tumor (EBV-SMT) [32]. Interestingly, both our patients had no evidence of EBV positivity and smooth muscle tumor mutation and only one of them had recurrent CMV infections, further highlighting the great variability of phenotypes. [33, 34]. Unfortunately, target therapy for CARMIL2 LOF is not available yet, and symptomatic therapy needs to be adjusted depending on clinical manifestations. Clinical reports describe mostly supportive and immunomodulatory treatments [13]. There are a few reports of successful allogeneic HSCT in CARMIL2 LOF patients, mostly at pediatric age [34, 35].

Mutations in STAT genes are notoriously associated with IEI, but there is not a known STAT gene mutation classified as a predominantly humoral defect according to the IUIS classification [4]. STAT3 LOF is considered causative of hyper-IgM syndrome, while STAT3 GOF gives regulatory T cells disorders [4]. Identifying mutations in STAT1 and STAT3, which are integral to the JAK-STAT signaling pathway, helps guiding specific therapeutic interventions aimed at modulating immune responses. There are reports of STAT1 GOF patients successfully treated with JAK inhibitors either as compassionate therapy or as bridging treatment in the peri-transplantation period [18, 36].

Despite almost ubiquitous ORAI1 expression, the channel has a nonredundant role in only a few cell types judging from the limited clinical phenotype in ORAI1-deficient patients [37]. Reports about ORAI1 null mutations describe reduced numbers of natural killer T and Treg cells that are likely for patients’ immune defects. Moreover, ORAI1-deficient patients have dental enamel defects and anhidrosis, representing a new form of anhidrotic ectodermal dysplasia with immunodeficiency [38]. To the best of our knowledge, unfortunately there is not any report regarding therapeutic possibilities for this type of defect.

Overall, TGP sequencing was crucial in detecting pathogenic variants in eight patients with monogenic immune defects. These genetic insights not only confirmed the diagnoses but also provided critical information for a better understanding of the genetic basis underlying their phenotype and for considering tailored treatment strategies. One of the major challenges in diagnosing IEIs is the phenotypic variability and the presence of overlapping syndromes. Data shown here illustrate how different genetic variants can lead to similar clinical presentations, complicating the diagnostic process. For example, both TACI and CARMIL2 variants were associated with recurrent infections and autoimmune manifestations, yet each required different management approaches. However, while NGS offers substantial benefits, its implementation in clinical practice is often limited by resource constraints which was also a limitation in this study. The high cost of sequencing and the need for specialized bioinformatic analysis can restrict access to this technology, particularly in resources-limited settings. The progressive affordability is likely to expand the use of NGS in the clinical setting, making genetic testing more accessible to a broader patient population. Meanwhile, we showcased how prioritizing specific clinical features—such as autoimmunity, autoinflammation, and syndromic traits—can optimize resource allocation and enhance diagnostic precision for the patients who need it most. Additionally, this approach contributes valuable insights and data to advance the complex journey of fully understanding and treating IEI.

## Data Availability

Reasonable requests for data sharing may be submitted to Antonio Giovanni Solimando as the corresponding author at antonio.solimando@uniba.it.
